# The Impact of Chatbot Response Strategies and Emojis Usage on Customers’ Purchase Intention: The Mediating Roles of Psychological Distance and Performance Expectancy

**DOI:** 10.3390/bs15020117

**Published:** 2025-01-23

**Authors:** Hua Meng, Xinyuan Lu, Jiangling Xu

**Affiliations:** 1 School of Information Management, Central China Normal University, Wuhan 430079, China; luxy@mail.ccnu.edu.cn; 2 Hubei E-Commerce Research Center, Central China Normal University, Wuhan 430079, China; 3 School of Fundamental Education, College of Arts and Sciences·Kunming, Kunming 650222, China

**Keywords:** artificial intelligence (AI), response strategies, emojis, chatbots, purchase intention, psychological distance, performance expectancy

## Abstract

Artificial intelligence (AI) chatbots have been widely adopted in customer service, playing a crucial role in improving service efficiency, enhancing user experience, and elevating satisfaction levels. Current research on the impact of chatbots on consumers’ purchase decisions primarily focuses on linguistic communication features, with limited exploration into the non-verbal social cues employed by chatbots. By conducting three scenario-based experiments, this study investigates the mechanisms through which chatbot response strategies (proactive vs. reactive) and the use of emojis (yes vs. no) influence users’ purchase intention. The findings suggest that proactive response strategies by chatbots are more effective in strengthening users’ purchase intention compared to reactive strategies. Psychological distance and performance expectancy serve as significant mediators in this relationship. Additionally, the use of emojis moderates the effect of chatbot response strategies on psychological distance, while its moderating effect on performance expectancy is not significant. This study offers new insights into non-verbal social cues in chatbots, revealing the psychological mechanisms underlying the influence of chatbot response strategies on users’ purchase decisions and contributing to the limited evidence on visual symbols as moderating factors. Furthermore, the findings provide practical recommendations for businesses on optimizing chatbot interaction strategies to enhance user experience.

## 1. Introduction

AI chatbots have become pivotal in revolutionizing customer service across various industries, including banking, marketing, and e-commerce. The continuous development and application of chatbots across various industries underscore their potential to revolutionize customer service operations (Haupt et al., 2023; Yu & Zhao, 2024). Chatbots are particularly well-suited for handling routine issues and simple requests, owing to their advantages of immediate response, automated interactions, 24/7 availability, the capacity to handle large volumes of requests, and standardized service. Chatbots are considered innovative tools for delivering automated customer assistance and improving service delivery (Abdallah et al., 2023). The integration of chatbots into online services has enhanced customer convenience, efficiency, and personalized experiences. Research has found that a significant portion of consumers prefer interacting with chatbots during the pre-purchase decision stage (Majeed & Kim, 2024), highlighting the importance of chatbots in influencing product or service selection decisions. Overall, chatbots have reshaped the frontline interface in customer service, offering a combination of personalization, efficiency, and automation to enhance user experiences and propel business growth.

According to the existing literature, the research on chatbots in customer service has almost exclusively focused on anthropomorphism and its impact on user experience (X. Li & Sung, 2021; Vu & Nguyen, 2022). Anthropomorphism not only emphasizes the extent to which chatbots are endowed with human-like attributes, such as names, avatars, or voices, which serve as visual and auditory cues (Crolic et al., 2021; Nguyen et al., 2023), but also encompasses advanced human-like attributes like perceptual and cognitive abilities (Buyukgoz et al., 2022; Han et al., 2023; Terblanche & Kidd, 2022). It further requires chatbots to exhibit interactive characteristics in service environments, including specific emotions (Chien et al., 2024; Lv et al., 2021) and distinct language styles (Y. Huang & Gursoy, 2024; Roy & Naidoo, 2021). While extensive research has explored how to maximize the value of chatbot agents by humanizing AI through visual, auditory, and communicative cues (Han et al., 2023), there is limited attention on non-verbal social cues in chatbots. Studies have found that non-verbal strategies also play a significant role in chatbot service interactions (Schanke et al., 2021). Among them, response strategies in chatbot service interactions are considered important non-verbal social cues (Keidar et al., 2024; D. Li et al., 2022). In e-commerce shopping platforms, intelligent service robots have become a vital component of the consumer experience. These robots are capable of providing proactive service to consumers immediately upon clicking the chat window. For instance, when a consumer browses a specific product and clicks for consultation, the intelligent service robot promptly auto-responds with detailed information about the product, its features, and any applicable promotional policies. Furthermore, based on consumers’ browsing history, purchase history, and other relevant information, these robots can actively recommend related products or promotional information to consumers. For example, if a consumer has previously purchased sports shoes, upon their next login to the shopping platform, the robot will proactively recommend related sports accessories or attire. These intelligent service robot response strategies are widely utilized on renowned Chinese e-commerce platforms such as Taobao, Tmall, and JD.com. However, existing research has not fully explored how specific response strategies (proactive vs. reactive) of chatbots affect users’ purchase intention, and the psychological mechanisms and boundary conditions underlying this effect remain to be uncovered. Consequently, a significant disparity persists between theoretical understanding and practical application.

Existing research has identified psychological distance and performance expectancy as psychological mechanisms through which chatbot interactions influence user behavior (Gursoy et al., 2019; X. Li & Sung, 2021; Lv et al., 2021, 2022; Park et al., 2024). It remains to be empirically tested whether psychological distance and performance expectancy also mediate the relationship between chatbot response strategies and user purchase intention. Additionally, research has indicated that emojis, due to their visual and emotional attributes, can be utilized in marketing activities to attract attention, stimulate social interaction, and significantly enhance consumer experience and purchase intention (Bai et al., 2019). The application scope of emojis has expanded from social media communication to customer service. Their function has shifted from primarily facilitating interpersonal emotional interactions to enhancing the affinity and efficiency of customer service within the marketing experience, thereby optimizing consumer experiences. For instance, customer service representatives use the “smiley face” emoji in greetings to express friendliness, the “sorry” emoji to convey apologies when customers are dissatisfied, the “OK hand” emoji to signify acknowledgment upon receiving user instructions, and the “smiling face with a bouquet of flowers” emoji to express pleasure in response to user gratitude. With the widespread adoption of chatbots in customer service, emojis have also been extensively incorporated into interactions facilitated by these automated agents. The role of emojis in moderating user perceptions and behaviors in chatbot service interactions is an emerging area with limited empirical evidence. This study aims to address these issues by systematically exploring the direct impact of chatbot response strategies, the mediating roles of psychological distance and performance expectancy, and the moderating effects of emojis, making a unique contribution to the comprehensive understanding of how these factors interact to influence user purchase intention. This study investigates the following questions:

- RQ1: How do chatbot response strategies (proactive/reactive) directly influence user purchase intention?
- RQ2: How do psychological distance and performance expectancy mediate the relationship between chatbot response strategies and purchase intention?
- RQ3: How do emojis moderate the impact of chatbot response strategies on psychological distance and performance expectancy?

This study makes two major theoretical contributions. Firstly, while previous research has primarily focused on the language strategies used by chatbots in service contexts, this study emphasizes the non-verbal strategies, specifically the proactive/reactive response strategies, that are employed by chatbots in service interactions. It reveals how these response strategies influence users’ purchase intention through psychological distance and performance expectancy. This enriches and deepens the understanding of non-verbal social cues and their mechanisms in chatbot services, providing new insights into human–machine interactions in the context of customer service. Secondly, this study contributes to the limited knowledge on the moderating factors in customer–chatbot interactions. The moderating role of emojis in chatbot interactions is an emerging area of research. By examining how emojis moderate the impact of chatbot response strategies on psychological distance and performance expectancy, this study provides preliminary empirical evidence, expanding the understanding of the role of emojis in human–chatbot interactions. Moreover, the findings offer theoretical foundations and practical guidance for businesses on how to optimize chatbot interaction strategies, enhance user experience, and increase purchase intention.

## 2. Literature Review

While AI-based chatbots offer opportunities to streamline service processes, save costs and time, and provide round-the-clock service (Blazevic & Sidaoui, 2022), their execution often falls short of meeting customer expectations for high-level agents (Crolic et al., 2021), leading to reduced user compliance (Adam et al., 2021). For example, chatbots often fail to deliver satisfactory responses to complex user requests, resulting in user dissatisfaction, negative word-of-mouth, and users abandoning chatbot interactions (Haupt et al., 2023). Despite this, chatbots are widely used to provide automated customer service, enhancing interactions between customers and brands (M.-H. Huang & Rust, 2021). Currently, the service industry is exploring the effectiveness of collaboration between human employees and digital employees, such as AI-driven chatbots. The concept of hybrid service agents, which combines chatbots with human agents, is emerging, leveraging the strengths of both in online services (Gnewuch et al., 2023). However, to fully leverage the advantages of chatbots in customer service, it is crucial to address consumer skepticism and ensure appropriate design and implementation.

Studies have emphasized the significance of integrating interactive user interface design elements into chatbots for optimizing user experience and retention (Ramesh & Chawla, 2022). Anthropomorphizing chatbots has been identified as a facilitator of effective customer interaction (Cai et al., 2022). Anthropomorphism can improve users’ perceptions (e.g., perceived intelligence), evaluations (e.g., usefulness), and relationships (e.g., rapport) with chatbots (Blut et al., 2021), thereby enhancing customer satisfaction and augmenting purchase intention (Klein & Martinez, 2023). Anthropomorphizing chatbots can foster user trust (Mostafa & Kasamani, 2022), ultimately enhancing the service quality of chatbots and contributing to increased satisfaction and retention (Q. Chen et al., 2023). Research has indicated that the communication strategies employed by chatbots are more pivotal than their profile design and error correction strategies (Zhou et al., 2024). Implementing anthropomorphic messaging strategies in chatbots enhances users’ social perception and interaction experience, making them feel as though they are interacting with a human-like entity, rather than merely a machine. The communication strategies of chatbots, encompassing both verbal and non-verbal social cues, play a crucial role in shaping consumer perceptions and satisfaction (Jiménez-Barreto et al., 2023). This is due to the fact that these factors influence the chatbot’s responsiveness. Responsiveness is key in chatbot communication, directly affecting customer satisfaction and indirectly influencing purchase intention (H. Jiang et al., 2022).

The existing research has primarily concentrated on the linguistic strategies of chatbots, and these research foci can be encapsulated as chatbot communication styles, encompassing language formality (M. Li & Wang, 2023), language concreteness (Y. Huang & Gursoy, 2024; Jiménez-Barreto et al., 2023), service scripts (Sands et al., 2021), social orientation (De Cicco et al., 2020; Maar et al., 2023), social presence (Tsai et al., 2021), politeness (Song et al., 2023), warmth (Roy & Naidoo, 2021), and humor (Shin et al., 2023) (The studies are summarized in Table 1). Apart from linguistic communication attributes, chatbots also employ non-verbal social cues during service interactions, which this paper designate as chatbot engagement strategies, such as communication delays (Schanke et al., 2021), typing indication, and delayed response typing speed (Zhou et al., 2024). Nevertheless, research pertaining to chatbot engagement strategies remains controversial and inadequate. Some researchers have argued that prioritizing the study of proactive and reactive interaction modes of chatbots in service systems is essential (Hao et al., 2017; D. Li et al., 2022). Research has indicated that chatbot service proactivity is positively related to customer engagement through their trust in service robots (D. Li et al., 2022). During interactions with chatbots that proactively offer services, users are inclined to assign higher trust ratings, rooted in their cognitive evaluation of the chatbot. This proactive approach significantly encourages users’ intention to continue interacting with chatbots in the future (Chien et al., 2024). In comparison to the reactive mode, the proactive mode amplifies user performance and comprehension of the system, while concurrently mitigating perceived workload (Keidar et al., 2024). Nonetheless, proactive service can lead to customer resentment and dissatisfaction, particularly when it exceeds customer expectations or is perceived as intrusive. Research indicates that, while proactive service can enhance customer satisfaction, it may also generate negative responses if customers feel overwhelmed or pressured by excessive engagement from service employees (Otterbring et al., 2024). For instance, some studies have highlighted that the timing and manner of proactive service significantly influence customer reactions, suggesting that poorly timed interventions can lead to feelings of service redundancy and annoyance (Otterbring et al., 2024; Tsaur & Yen, 2019). There is empirical evidence suggesting that automatically initiated chatbots (that is, unsolicited advice from chatbots, as contrasted with user-prompted reactive responses) may elicit heightened psychological reactance, subsequently contributing to increased choice difficulty (Pizzi et al., 2021). Consequently, further investigation is imperative to delve into the effects of chatbot engagement strategies, specifically proactive and reactive response strategies, on user attitudes and behaviors, to provide deeper insights into the chatbot–customer relationship.

In the research on chatbot service interactions, psychological distance and performance expectancy are frequently considered as mediating mechanisms that influence user attitudes and behaviors during human–chatbot interactions. These studies have primarily concentrated on how anthropomorphism and human-like representations in chatbots impact users’ attitudes and behaviors through psychological distance and performance expectancy, exemplified by users’ evaluations of chatbots (X. Li & Sung, 2021), tolerance towards assistant service failures (Lv et al., 2021), acceptance of AI devices (Gursoy et al., 2019), intentions to utilize AI applications (Lv et al., 2022), and compliance intentions (Park et al., 2024). Psychological distance pertains to users’ emotional connection and trust in chatbots. Performance expectancy encapsulates users’ expectations regarding a chatbot’s perceived ability, perceived usefulness, and perceived ease of use. Psychological distance and performance expectancy influence users’ attitudes and behaviors by shaping their cognitive and emotional experiences (Schelble et al., 2023). The question of whether psychological distance and performance expectancy mediate between specific non-verbal social cues (including proactive/reactive strategies) and users’ purchase decisions remains open and is worth exploring. In customer service, non-verbal social cues exhibited by service personnel significantly influence customers’ psychological and performance perceptions. Research indicates that positive non-verbal behaviors, such as facial expressions and proactive engagement, enhance customer perceptions of service quality and foster emotional connections, leading to increased customer satisfaction and loyalty (Liang et al., 2020; Olk et al., 2021). Proactive AI systems, such as chatbots, can anticipate customer needs, leading to a more seamless experience that positively influences customer emotions and perceptions of service quality (D. Li et al., 2022; S. Q. Liu et al., 2024). This proactive engagement not only enhances customer satisfaction, but also encourages positive behavioral intentions, such as repurchase and word-of-mouth recommendations (S. Chen et al., 2023; El-Shihy et al., 2024) These research findings provide traceable evidence to support the exploration in this paper of the psychological mechanisms through which chatbot response strategies influence customers’ purchase intentions.

Additionally, the media richness theory and computer-mediated communication theory posit that visual symbols, such as emojis, can markedly improve user experience in digital communication by conveying emotions and increasing interaction richness (Bai et al., 2019; Erle et al., 2022). Among the diverse anthropomorphic cues utilized in chatbots, including language, turn-taking, politeness, and interactivity, the employment of emojis prominently stands out and significantly influences users’ social and emotional reactions towards chatbots (Chung & Han, 2022). Several studies have advocated that the utilization of emojis in chatbots is gaining prominence and recommend their adoption in chatbots (Nguyen et al., 2023; Yu & Zhao, 2024). Nevertheless, research focusing on the application of emojis in chatbots remains relatively limited, particularly in terms of empirical investigations into the efficacy of emojis in chatbot service interactions (Nguyen et al., 2023). In certain empirical studies, emojis have often been utilized solely as experimental manipulations to vary chatbot anthropomorphism levels (Chung & Han, 2022). Some research has empirically demonstrated that emojis exert direct effects on perceived capabilities and user engagement. Additionally, other studies have empirically analyzed the indirect effect of emojis on service satisfaction through enhancing the perceived warmth of chatbots (Yu & Zhao, 2024). In research on chatbot service failure recovery strategies, humorous emojis have been shown to increase consumers’ reuse intention by enhancing their perceived intelligence (D. Liu et al., 2023). The moderating role of emojis in enhancing the effectiveness of chatbot services remains largely unexplored. Consequently, it is of paramount importance to investigate the moderating role of emojis in the relationship between AI chatbot response strategies and users’ purchase intentions.

## 3. Hypothesis Development

### 3.1. Chatbot Response Strategies and Users’ Purchase Intention

In the past, the majority of chatbot interactions with humans have been heavily reliant on human initiative, where users initiate requests and chatbots subsequently generate and execute plans to fulfill them. This mode of interaction is commonly referred to as reactive behavior or passive service (Hao et al., 2017). This approach tends to be overly mechanical, failing to adequately satisfy the demand for a more human-like interaction experience. Recently, a proactive service model for service robots has emerged, incorporating both surface communication information and deep cognitive insights. These service robots anticipate user intentions and preferences by drawing inferences from user behavior and potential future scenarios, thereby offering proactive services (Buyukgoz et al., 2022; Grosinger et al., 2019). Put simply, information is proactively “pushed” to users without their explicit request, sometimes even preceding their initial inquiry. Proactive services have the capacity to discern user motivations, facilitating personalized and real-time service delivery.

A proactive response strategy enhances user satisfaction and trust by anticipating user needs and offering tailored suggestions. Conversely, a reactive response strategy entails chatbots responding solely after users have posed a question or made a request (Hao et al., 2017). While this strategy may still fulfill user needs, it often lacks initiative and personalization, potentially resulting in a less engaging and thoughtful service experience, which could diminish users’ purchase intention (Oh et al., 2021). Proactive chatbots facilitate greater user engagement, elicit more meaningful responses, and consequently enhance the overall user experience (Xiao et al., 2020). In summary, proactive responses lead to higher levels of satisfaction and a superior user experience compared to reactive responses (Pizzi et al., 2021). Studies have confirmed a positive correlation among chatbot proactivity, trust, and customer engagement (Chien et al., 2024; D. Li et al., 2022), contributing to the cultivation of customer purchase intention. Furthermore, research has underscored that proactive responses can steer users towards suitable options, ultimately enhancing their purchase intention (Ischen et al., 2020). Consequently, the following hypothesis is formulated:

**H1.**  *There is a significant difference in the impact of different chatbot response strategies on users’ purchase intention. Compared to reactive responses, proactive responses are more effective in enhancing users’ purchase intention.*

### 3.2. The Mediating Role of Psychological Distance

Psychological distance refers to the perceived and cognitive distance between an individual and an object or another person. This concept encompasses multiple dimensions, including temporal, spatial, social, and hypothetical, all of which influence perception and decision-making (Trope & Liberman, 2000). As psychological distance decreases, perceptions become more concrete and immediate, subsequently influencing behavior and decision-making (Trautmann, 2019). Research has established that psychological distance functions as a mediator between perception and behavior (F. Xu et al., 2020). There is evidence that psychological distance mediates the interactions between users and AI assistants, particularly when anthropomorphism is employed, as it reduces the perceived distance (X. Li & Sung, 2021). Moreover, research has illuminated the serial mediation effect of psychological distance and trust on compliance intentions within human–chatbot interactions, highlighting the mechanism through which psychological distance impacts user behavior (Park et al., 2024). Consequently, it is plausible to posit that psychological distance functions as a pivotal mediator between human–chatbot interactions and users’ purchase intentions.

Proactive service responses can bridge the psychological gap between service providers and their customers. In chatbot service interactions, proactive response strategies can mitigate psychological distance across various dimensions, encompassing time, space, social, and hypothetical distances. Proactive responses deliver information promptly, reducing the perceived waiting time for users (Q. Chen et al., 2023). Chatbots equipped with proactive response capabilities can transcend spatial limitations, subsequently reducing the perceived spatial distance between users and service providers (S. Chen et al., 2023; Ghosh et al., 2024; Matini et al., 2024). Personalized interactions facilitated by proactive response strategies often elicit emotional resonance in users, fostering stronger social bonds between users and chatbots, thereby mitigating social distance (B. Li et al., 2023; Wang et al., 2022). Furthermore, proactive response strategies present tailored and pertinent solutions, enhancing the authenticity and relevance of the service for users, ultimately mitigating hypothetical distance (Aslam, 2023; Trzebiński et al., 2023). Collectively, proactive response strategies encourage user–chatbot engagement and potentially foster a psychological reliance on the chatbot (Xie et al., 2023), ultimately bolstering users’ purchase intentions. Thus, we propose the following hypothesis:

**H2.**  *Psychological distance mediates the effect of chatbot response strategies on users’ purchase intention. Compared to reactive response strategies, the proactive response strategies of chatbots decrease users’ psychological distance and subsequently increase their purchase intention.*

### 3.3. The Mediating Role of Performance Expectancy

Performance expectancy refers to an individual’s perception of how the adoption of a new technology will enhance their job performance (Eiskjær et al., 2023). In the context of chatbot services, performance expectancy relates to customers’ expectations concerning the reliability and consistency of the services offered by chatbots (Gursoy et al., 2019; Lv et al., 2021). Several studies have highlighted that performance expectancy serves as the most potent predictor of behavioral intention when compared to other variables, such as effort expectancy and facilitating conditions (Azalan et al., 2022; J. Kim & Lucas, 2024). Moreover, performance expectancy has been demonstrated to have a positive impact on diverse outcomes (Milenia Ayukharisma & Budi Santoso, 2024), including user acceptance and behavioral intentions (Paraskevi et al., 2023; Terblanche & Kidd, 2022; Tian et al., 2024). It has been observed that performance expectancy significantly bolsters users’ initial trust in chatbots, subsequently leading to an increase in their intention to utilize chatbots and fostering user engagement (Mostafa & Kasamani, 2022). Collectively, these studies offer profound insights into the positive correlation between performance expectancy and users’ behavioral intentions towards utilizing chatbots.

Performance expectancy is typically reflected by factors such as perceived usefulness and perceived ease of use (Lv et al., 2022). In other words, the adoption and acceptance of chatbots are influenced by factors including perceived usefulness and perceived ease of use (Myin & Watchravesringkan, 2024). Research has demonstrated that the perceived usefulness and perceived ease of use of chatbots play a pivotal role in enhancing customer acceptance and purchase intention (Abdallah et al., 2023). Furthermore, factors such as customer engagement and continuance intention are also influenced by the perceived performance and utility offered by chatbots (B. Li et al., 2023). The personalization and efficiency of the service, alongside interactivity and information quality, contribute to enhancing the perceived usefulness of chatbots (Pillai et al., 2024). Research has shown that information quality positively influences perceived usefulness and perceived ease of use, both of which are crucial factors driving customer trust, attitudes, and behavioral intentions (Kang & Namkung, 2019). This evidence indicates that perceived usefulness and perceived ease of use mediate the relationships between interactivity, information quality, customer trust, and purchase intention (Zhu et al., 2023b). Consequently, proactive response strategies can bolster users’ performance expectancy, ultimately stimulating their purchasing behavior. Therefore, the following hypothesis is formulated:

**H3.**  *Performance expectancy mediates the effect of chatbot response strategies on users’ purchase intention. Compared to reactive response strategies, proactive response strategies of chatbots amplify users’ performance expectancy and subsequently increase their purchase intention.*

### 3.4. The Moderating Role of Emojis

The media richness theory posits that the richness of a medium affects communication effectiveness, with the richer the medium, the better it can convey complex information and emotions. Emojis, as a visually rich form of media, enrich user experience and foster psychological connection by infusing interactions with an emotional dimension. Emojis are visual symbols that play a crucial role in computer-mediated communication (CMC), serving as non-verbal cues that evoke emotional responses in digital interactions, similar to facial expressions in face-to-face communication. They serve as a potent tool for conveying emotions within online environments, making up for the absence of emotional cues beyond body language and vocal inflections in chatbots (D. Liu et al., 2023). Including emojis in messages can improve users’ emotional states, providing personalized and friendly content compared to plain text (Beattie et al., 2020). Research has demonstrated that emojis augment the emotional intensity of messages in digital communication, raising their emotional value and influencing behavior through inference and emotional reactions (Erle et al., 2022). The sociological theory of emotion work suggests that emojis, as tools for conveying emotions, facilitate the maintenance and enhancement of social relationships (Riordan, 2017).

Emojis not only elicit emotional responses in individuals, but also significantly influence social cognition. Emojis have been employed to investigate online trust among users, underscoring their influence on interpersonal relationships. In chatbot service interactions, the inclusion of emojis in chatbot responses serves as cues for perceived anthropomorphism, subsequently affecting users’ perceived psychological distance (Nguyen et al., 2023; Park et al., 2024). Studies have demonstrated that the use of emojis in chatbot conversations can enhance perceived warmth (Yu & Zhao, 2024) and social attractiveness (Beattie et al., 2020). Prior research has indicated that chatbots employing emojis are capable of fostering user trust and diminishing perceived psychological distance. Proactive response strategies offer efficient and tailored service, with the utilization of emojis further infusing an emotional aspect into interactions, thereby fostering a stronger psychological bond with users. In reactive response strategies, the incorporation of emojis can partly offset the absence of proactivity by enriching the emotional aspect of communication, subsequently diminishing psychological distance. Based on the aforementioned findings, the following hypothesis is formulated:

**H4.**  *Emojis moderate the relationship between chatbot response strategies and users’ psychological distance. Compared to not using emojis, chatbots with proactive responses that use emojis result in a smaller psychological distance. Conversely, chatbots with reactive responses that do not use emojis result in a larger psychological distance compared to those that use emojis.*

As non-verbal cues, emojis facilitate information transmission, capture recipients’ attention, enhance the vitality of communication, mitigate ambiguity in discourse, mold message comprehension, and ultimately optimize conversational outcomes (Ischen et al., 2020; Riordan, 2017; Wu et al., 2022). Researchers have emphasized the role of chatbots in enhancing user engagement, arguing that emojis augment user experience and interaction (Beattie et al., 2020; Fournier-Tombs & McHardy, 2023; Wang et al., 2022). The presence or absence of emojis can also influence the credibility and competence attributed to chatbots, subsequently affecting user trust and acceptance (Youn & Cho, 2023). Emojis used by chatbots can enhance customers’ intentions for continued use in service recovery scenarios (Zhou et al., 2024; Zhu et al., 2023b). Enhancing users’ trust, engagement, acceptance, and intention to continue using chatbots leads to higher performance expectancy. Consequently, it is plausible to contend that for proactive chatbots, the incorporation of emojis further augments the interaction experience, thereby fostering heightened performance expectancy among users. Notwithstanding the inherent lack of proactivity in reactive response strategies, the employment of emojis can enrich the depth and pleasure of interactions, subsequently augmenting users’ intentions to persist in usage and enhancing their performance expectancy. Hence, the subsequent hypothesis is formulated:

**H5.**  *Emojis moderate the relationship between chatbot response strategies and users’ performance expectancy. Compared to not using emojis, chatbots with proactive responses that use emojis elicit a stronger performance expectancy. Conversely, chatbots with reactive responses that do not use emojis elicit a weaker performance expectancy compared to those that use emojis.*

**Figure 1:**
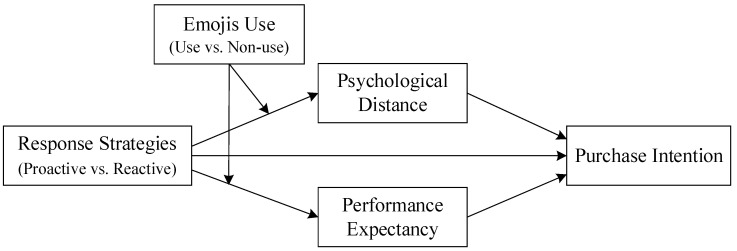
Theoretical model of this study.

Based on the above hypotheses, the following theoretical model (Figure 1) is constructed for this study:

## 4. Method and Results

This study collected data via three experiments to test the formulated hypotheses. A method utilizing situational experiments that integrate text and images was employed, which has been widely adopted and recognized as effective. Specifically, participants were instructed to meticulously review both textual and pictorial materials to gain a deeper understanding of the experimental scenarios and envision themselves within the described contexts, subsequently completing pertinent questionnaires. The textual materials detailed the experimental scenarios, whereas the pictorial materials comprised screenshots showcasing the chatbot Tina engaging with customers. To mitigate the potential for random effects arising from experimental scenarios, the three experiments adopted distinct situational setups. These setups encompassed online retail shopping (Study 1) as well as online hotel room reservations (Study 2 and 3).

In all three studies, pilot experiments were conducted prior to the formal experiments to verify the validity of the experimental materials. Study 1 employed a single-factor between-subjects experimental design to examine the impact of chatbot response strategies (proactive vs. reactive) on users’ purchase intentions (i.e., to test H1). Study 2 also utilized a single-factor between-subjects experimental design to investigate the mediating roles of psychological distance and performance expectancy in the relationship between chatbot response strategies and users’ purchase intentions (i.e., to test H2 and H3). Study 3 adopted a two-factor between-subjects experimental design with two levels each for response strategy (proactive vs. reactive) and emojis use (yes vs. no), to examine the moderating effect of emojis (i.e., to test H4 and H5).

### 4.1. Study 1

In Study 1, the experimental scenario centered on customers engaging in online clothing shopping. Participants were instructed to envision themselves in a situation wherein they were required to purchase a blue business shirt and explored clothing options on an e-commerce platform. They posed inquiries about the shirt through the platform’s customer service chatbot, Tina. The recruited participants were randomly assigned to two distinct groups. The textual descriptions furnished to both groups remained identical, whereas the screenshots depicting Tina’s interactions with customers differed. Apart from the varying response strategies adopted by the chatbot, all other visual elements present in the two screenshots were consistent. The proactive chatbot provided product introductions and purchase suggestions based on the customer’s browsing history, even before the customer posed any inquiries. In contrast, the reactive chatbot introduced products step-by-step based on the customer’s questions, and only offered purchase suggestions after the customer requested them.

#### 4.1.1. Pilot Test

A pre-study was conducted on Credamo (https://www.credamo.com), a Chinese online survey platform similar to MTurk, where 60 participants were recruited. Participants were randomly assigned to one of two groups. After reading the experimental materials, participants were asked to answer the following two questions: (1) To what extent do you perceive the chatbot as proactive in responding to customer inquiries? (2) To what extent do you perceive the chatbot as reactive in responding to customer inquiries? Their responses were gauged using a 7-point Likert scale, with 1 representing “strongly disagree” and 7 signifying “strongly agree”. This methodology was employed consistently in all subsequent experiments.

A *t*-test yielded statistically significant differences between the two experimental groups, demonstrating that participants in the proactive response group perceived a significantly higher level of proactivity compared to those in the reactive response group (i.e., for the first question: M__proactive_ = 5.60, M__reactive_ = 2.90, t = 14.100, *p* < 0.001). In contrast, participants in the reactive response group perceived a significantly higher level of reactivity in comparison to those in the proactive response group (i.e., for the second question: M__proactive_ = 3.23, M__reactive_ = 5.33, t = −10.944, *p* < 0.001). As a result, participants accurately discerned the response strategies utilized by the chatbots, wherein participants in the proactive (vs. reactive) response group perceived Tina as proactive (vs. reactive). This observation underscores the successful fulfillment of the manipulation requirements by the experimental materials.

#### 4.1.2. Procedure and Measures

The main experiment employed the identical materials and manipulation procedures as those utilized in the pre-study. A total of 160 participants were recruited. The dependent variable, purchase intention, was assessed using a scale adapted from previous studies (H. Jiang et al., 2022; Roy & Naidoo, 2021; Sands et al., 2021), with three items: “If I were to purchase this product, I might consider buying it from this platform”, “If I were to purchase this product, I would be willing to buy it from this platform”, and “If I were to purchase this product, I would be prepared to buy it from this platform”. The independent variable was operationalized using methodologies drawn from the existing literature (Roy & Naidoo, 2021; Song et al., 2023), with two items: “The chatbot Tina proactively responds to customer inquiries” and “The chatbot Tina reactively responds to customer inquiries”.

Following the exclusion of 21 invalid questionnaires, the effective sample size for the main experiment was reduced to 139, yielding an efficiency rate of 86.88%. The proactive response group comprised 73 participants, whereas the reactive response group consisted of 66 participants. The sample included 73 males (52.52%) and 66 females (47.48%). In terms of educational background, the sample comprised 29 participants holding a college diploma (20.86%), 76 with a bachelor’s degree (54.68%), and 34 with a postgraduate degree (24.46%). The *t*-test demonstrated a statistically significant difference between the two experimental groups (i.e., for the first question: M__proactive_ = 5.68, M__reactive_ = 3.00, t = 20.697, *p* < 0.001; for the second question: M__proactive_ = 3.14, M__reactive_ = 5.38, t = −16.611, *p* < 0.001). Consequently, the manipulation of the independent variable was deemed successful. The scales for purchase intention demonstrated good reliability and validity, with Cronbach’s α = 0.928 and KMO = 0.744, both surpassing the commonly accepted threshold of 0.7.

#### 4.1.3. Results

**Figure 2:**
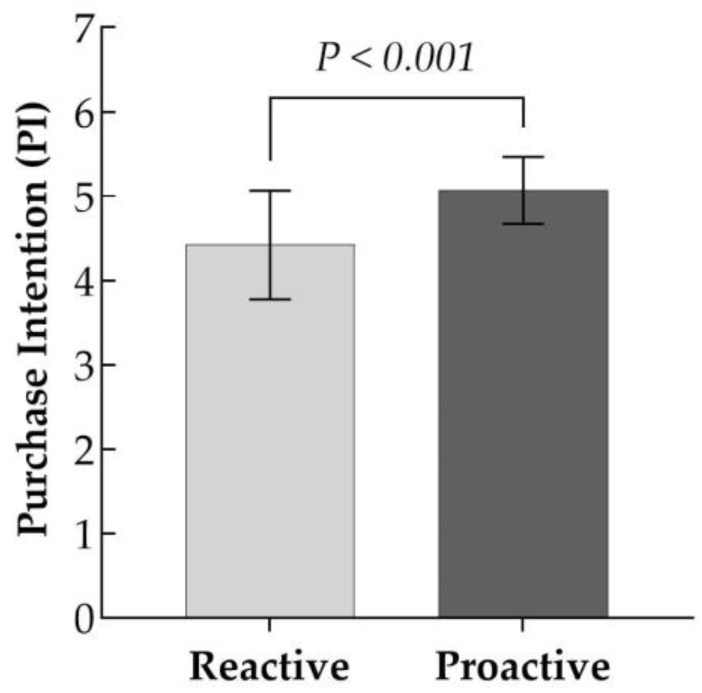
Impact of chatbot response strategies on users’ purchase intention (Study 1).

Using a one-way ANOVA, the study empirically tested the impact of different chatbot response strategies (proactive vs. reactive) on users’ purchase intentions. As depicted in Figure 2, the data analysis results indicate a statistically significant difference in purchase intentions between the two experimental groups (M__proactive_ = 5.070, M__reactive_ = 4.422, F(1, 137) = 51.359, *p* < 0.001). This finding suggests that chatbot proactive response strategies are more efficacious in bolstering customer purchase intentions when compared to reactive response strategies, thereby corroborating H1.

### 4.2. Study 2

Study 2 adhered to the same experimental procedure as Study 1, with the exception of altering the experimental context to an online hotel booking scenario. Specifically, customers intended to book a hotel room through a hotel reservation platform. After browsing the hotel rooms, they inquired about relevant information from the intelligent service robot Tina. The proactive and reactive response modes of the chatbot presented in the screenshots were the same as those in Study 1.

#### 4.2.1. Pilot Test

In the pilot study, a total of 60 participants were recruited and then randomly allocated to two separate groups. After reviewing the experimental materials, participants responded to judgmental questions pertaining to both proactive and reactive strategies, mirroring those posed in the pre-experiment of Study 1. The results of the *t*-test demonstrated that the experimental materials met the manipulation requirements (for the first question: M__proactive_ = 5.48, M__reactive_ = 3.31, t = 13.093, *p* < 0.001; for the second question: M__proactive_ = 3.74, M__reactive_ = 5.14, t = −9.870, *p* < 0.001).

#### 4.2.2. Procedure and Measures

A total of 160 participants were recruited. The measurement methods for the dependent and independent variables were consistent with those used in Study 1, and additional measures for psychological distance and performance expectancy were included. The measurement of psychological distance was adapted from previous research (X. Li & Sung, 2021; Park et al., 2024) and comprised three items: “I feel familiar with the chatbot”, “I feel a sense of closeness with the chatbot”, and “I think the chatbot can be someone I can be friends with”. The measurement of performance expectancy was derived from pertinent studies (Gursoy et al., 2019; Lv et al., 2021, 2022) and comprised four items: “I find the chatbot useful”, “Using the chatbot helps me find solutions faster”, “Using the chatbot makes me feel more capable”, and “I believe the chatbot helps solve problems”.

After excluding 18 invalid questionnaires, the final sample size was 142, with a valid response rate of 88.75%. The proactive response group comprised 68 participants, and the reactive response group comprised 74 participants. Among the valid samples, there were 81 males (57.04%) and 61 females (42.96%). The educational background distribution was as follows: 23 participants with a college diploma (16.19%), 84 with a bachelor’s degree (59.15%), and 35 with a postgraduate degree (24.65%). The *t*-test results revealed the successful manipulation of the independent variable (for the first question: M__proactive_ = 5.50, M__reactive_ = 3.35, t = 19.241, *p* < 0.001; for the second question: M__proactive_ = 3.62, M__reactive_ = 5.28, t = −14.774, *p* < 0.001). The scales for psychological distance (Cronbach’s α = 0.842, KMO = 0.721), performance expectancy (Cronbach’s α = 0.881, KMO = 0.831), and purchase intention (Cronbach’s α = 0.817, KMO = 0.718) exhibited good reliability and validity.

#### 4.2.3. Results

The one-way ANOVA results (as shown in Table 2) indicated a statistically significant difference in psychological distance between the two experimental groups (M__proactive_ = 4.853, M__reactive_ = 4.054, F(1, 140) = 94.730, *p* < 0.001), suggesting that proactive response strategies are more effective at improving users’ psychological distance than reactive strategies. Likewise, the proactive response group exhibited higher performance expectancy scores than the reactive response group (M__proactive_ = 4.787, M__reactive_ = 3.986, F(1, 140) = 78.902, *p* < 0.001), indicating the effectiveness of proactive response strategies in elevating users’ performance expectancy. Furthermore, a significant difference in purchase intention was observed between the two groups (M__proactive_ = 5.186, M__reactive_ = 4.495, F(1, 140) = 83.471, *p* < 0.001), aligning with the results from Study 1 and reinforcing support for H1.

The independent variable was set as a dummy variable, with the reactive response strategy coded as 0 and the proactive response strategy coded as 1. The bootstrap method was utilized to examine the mediating effects of psychological distance and performance expectancy on purchase intention. The results (as shown in Table 3) revealed a significant direct effect of chatbot response strategy on purchase intention (β__direct_ = 0.237, LLCI = 0.075, ULCI = 0.398, the 95% confidence interval does not include 0). Both the indirect effects of psychological distance (β__indirect_ = 0.221, BootLLCI = 0.048, BootULCI = 0.388, excluding 0) and performance expectancy (β__indirect_ = 0.234, BootLLCI = 0.086, BootULCI = 0.398, excluding 0) were found to be statistically significant. Consequently, hypotheses H2 and H3 are supported.

### 4.3. Study 3

The manipulation procedures and experimental scenario in Study 3 mirrored those employed in Study 2. Nonetheless, the manipulation materials in Study 3 diverged, comprising four screenshots showcasing chat interactions between the chatbot and a customer: (I) proactive response without emojis; (II) reactive response without emojis; (III) proactive response with emojis; (IV) reactive response with emojis. Screenshots (I) and (II) represented the manipulation materials utilized in Study 2, whereas screenshots (III) and (IV) were modified versions of (I) and (II), respectively, with the addition of emojis. The emojis used were all universally recognized, and the emojis used in the two screenshots remained consistent.

#### 4.3.1. Pilot Test

The pre-test was designed to evaluate the efficacy of manipulating the independent variable in the presence of emojis. A total of 60 participants were recruited and subsequently randomly allocated to two distinct groups. The participants were instructed to review the experimental materials incorporating emojis and subsequently respond to the judgmental questions related to both proactive and reactive strategies, analogous to those posed in the pre-experiment of Study 1. The results of the *t*-test demonstrated statistically significant differences between the two groups. For the first question, M__proactive_ = 5.23, M__reactive_ = 3.90, t = 12.395, *p* < 0.001; for the second question, M__proactive_ = 3.60, M__reactive_ = 5.40, t = −11.755, *p* < 0.001. Consequently, the inclusion of emojis in the chatbot interaction screenshots did not hinder the effectiveness of the experimental materials in manipulating the independent variable.

#### 4.3.2. Procedure and Measures

A total of 340 participants were recruited. The methodologies employed for measuring the dependent variable, independent variable, and mediating variables mirrored those utilized in Study 2. Following the exclusion of 38 invalid questionnaires, the final sample size was reduced to 302, yielding an effective response rate of 88.82%. The sample sizes for the experimental groups were distributed as follows: Group I (proactive response without emojis) = 76, Group II (reactive response without emojis) = 76, Group III (proactive response with emojis) = 73, and Group IV (reactive response with emojis) = 77. Of the 302 valid samples, 149 were male (49.34%), and 153 were female (50.66%). In terms of educational background, there were 50 participants with a college diploma (16.56%), 180 with a bachelor’s degree (59.61%), and 72 with a postgraduate degree (23.84%). Additionally, the study investigated the usage of emojis and revealed that 85 participants occasionally utilized emojis (28.15%), whereas 217 participants frequently employed them (71.85%).

The *t*-test results indicated the successful manipulation of the independent variable, irrespective of the inclusion of emojis. Specifically, in the absence of emojis (Groups I and II), statistically significant differences were observed between the proactive and reactive response groups (for the first question: M__proactive_ = 5.67, M__reactive_ = 2.96, t = 22.111, *p* < 0.001; for the second question: M__proactive_ = 3.16, M__reactive_ = 5.33, t = −16.827, *p* < 0.001). Similarly, in the presence of emojis (Groups III and IV), statistically significant differences were also observed between the proactive and reactive response groups (for the first question: M__proactive_ = 5.34, M__reactive_ = 3.78, t = 18.964, *p* < 0.001; for the second question: M__proactive_ = 3.22, M__reactive_ = 5.03, t = −15.192, *p* < 0.001). The scales assessing psychological distance (Cronbach’s α = 0.844, KMO = 0.727), performance expectancy (Cronbach’s α = 0.856, KMO = 0.779), and purchase intention (Cronbach’s α = 0.876, KMO = 0.733) exhibited strong reliability and validity.

#### 4.3.3. Results

The one-way ANOVA results (as shown in Table 4) revealed that, irrespective of emojis usage, proactive chatbots consistently exhibited better psychological distance (with emojis: M__proactive_ = 5.639, M__reactive_ = 3.961, F(1, 148) = 440.068, *p* < 0.001; without emojis: M__proactive_ = 4.930, M__reactive_ = 3.667, F(1, 150) = 184.091, *p* < 0.001), higher performance expectancy (with emojis: M__proactive_ = 4.877, M__reactive_ = 3.481, F(1, 148) = 272.934, *p* < 0.001; without emojis: M__proactive_ = 4.829, M__reactive_ = 3.661, F(1, 150) = 143.639, *p* < 0.001), and elicited greater purchase intention (with emojis: M__proactive_ = 5.881, M__reactive_ = 4.251, F(1, 148) = 429.216, *p* < 0.001; without emojis: M__proactive_ = 4.952, M__reactive_ = 3.811, F(1, 150) = 161.106, *p* < 0.001). Bootstrapping-based mediation analysis indicated that both psychological distance (with emojis: 95% CI = [0.251, 0.791], excluding 0; without emojis: 95% CI = [0.504, 1.057], excluding 0) and performance expectancy (with emojis: 95% CI = [0.362, 0.833], excluding 0; without emojis: 95% CI = [0.095, 0.619], excluding 0) significantly mediated the relationship between chatbot response strategy and purchase intention. Thus, H1, H2, and H3 were confirmed once again.

**Figure 3:**
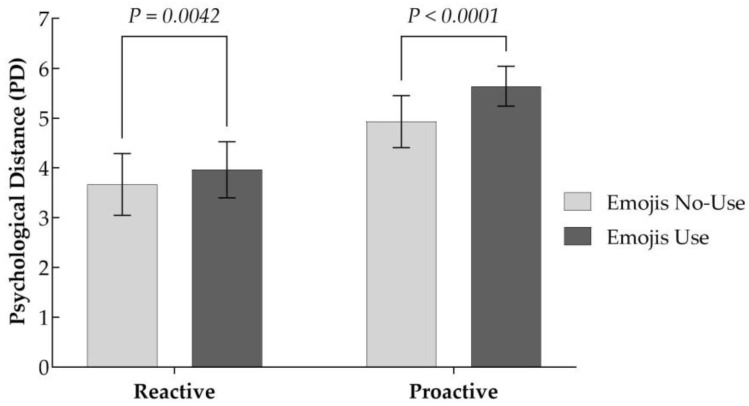
Moderating role of emojis in the relationship between RS and PD.

Analogous to the manipulation of the independent variable, the use of emojis is operationalized as a dummy variable, where “0” denotes the absence of emojis and “1” indicates the presence of emojis. The two-way ANOVA results, with psychological distance as the dependent variable (as shown in Table 5), indicated a significant interaction effect between chatbot response strategy and emojis usage (F = 11.410, *p* = 0.001 < 0.01), suggesting that the impact of chatbot response strategy on psychological distance is moderated by emoji usage. Specifically, as depicted in Figure 3, proactive chatbots utilizing emojis elicited superior psychological distance among users compared to proactive chatbots without emojis usage (M__with_emojis_ = 5.639, M__without_emojis_ = 4.930, *p* < 0.001). Conversely, reactive chatbots without emojis usage resulted in inferior psychological distance compared to reactive chatbots utilizing emojis (M__with_emojis_ = 3.961, M__without_emojis_ = 3.667, *p* < 0.01). Thus, H4 is confirmed.

**Figure 4:**
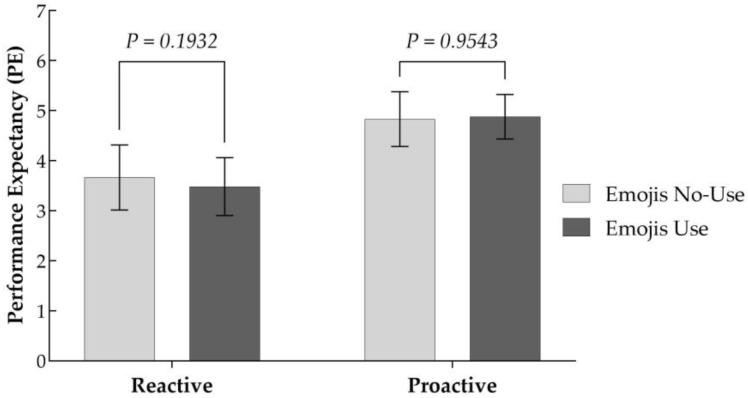
Moderating role of emojis in the relationship between RS and PE.

The two-way ANOVA results, with performance expectancy as the dependent variable (as shown in Table 6), indicate that the interaction effect between chatbot response strategy and emojis usage on performance expectancy is not significant (F = 3.130, *p* = 0.078 > 0.05). This finding implies that emojis do not significantly moderate the influence of chatbot response strategy on performance expectancy. Specifically, as depicted in Figure 4, proactive chatbots utilizing emojis did not significantly improve performance expectancy in comparison to proactive chatbots without emojis usage (M__with_emojis_ = 4.877, M__without_emojis_ = 4.829, *p* > 0.05). Likewise, reactive chatbots without emojis usage did not significantly diminish performance expectancy, in comparison to reactive chatbots utilizing emojis (M__with_emojis_ = 3.481, M__without_emojis_ = 3.661, *p* > 0.05). The findings presented in Figure 4 indicate that, albeit not statistically significantly, the use of emojis by reactive chatbots appears to have resulted in weaker performance expectancy. Consequently, H5 is not supported.

## 5. Conclusions and Discussion

### 5.1. Research Findings

Given that prior research on chatbot service interaction strategies has examined how linguistic strategies affect user purchase decisions (Jiménez-Barreto et al., 2023; Roy & Naidoo, 2021; Sands et al., 2021), we shift our focus to the non-verbal strategies employed in chatbot service interactions, particularly the response strategies of chatbots. This study delves into the mechanisms through which chatbot response strategies influence user purchase intention, explores the mediating roles of psychological distance and performance expectancy, and examines the moderating role of emojis. The findings indicate that: (i) in contrast to the reactive response strategy, a proactive response strategy markedly enhances user purchase intention (Study 1, supporting H1); (ii) psychological distance and performance expectancy mediate the above relationships, and proactive response strategies enhance users’ psychological distance and performance expectancy (Study 2, supporting H2 and H3); (iii) emojis significantly moderate the effect of chatbot response strategies on psychological distance, yet do not moderate the effect on performance expectancy (Study 3, supporting H4 but not H5).

### 5.2. Theoretical Contribution

Firstly, by deepening the understanding of chatbots’ non-verbal social cues and their underlying mechanisms, this study provides novel insights into human–chatbot interaction within digital commerce services. Previous research has given lesser attention to non-verbal strategies employed in chatbot service interactions, specifically chatbots’ engagement strategies. A limited number of studies have examined the influence of proactive/reactive interactions on user trust, satisfaction, and willingness to continue use (Chien et al., 2024; D. Li et al., 2022; Pizzi et al., 2021). This study extends these research endeavors by broadening the scope of predicted variables to include users’ purchase intentions, underscoring the importance of proactive response strategies in chatbot service interactions. The technology acceptance model (TAM) suggests that perceived ease of use and perceived usefulness significantly influence users’ intentions to adopt technology, including chatbots (Yoon & Yu, 2022). By providing proactive assistance, chatbots can enhance perceived usefulness, as they anticipate user needs and offer timely information, which can lead to increased purchase intentions (Zhu et al., 2023a). Moreover, the expectation confirmation theory (ECT) posits that users’ satisfaction is influenced by the confirmation of their expectations. Proactive chatbots that exceed user expectations can foster greater satisfaction, which in turn enhances loyalty and purchase intentions (Meyer et al., 2022; Y. Xu et al., 2022b). Additionally, the emotional engagement facilitated by proactive interactions can create a sense of connection, further driving purchase behavior (Y. Xu et al., 2022b). Thus, integrating proactive strategies in chatbot design not only improves user experience but also aligns with established theories that underscore the importance of user satisfaction in driving consumer behavior.

Secondly, this study elucidates the mechanisms by which chatbot response strategies influence users’ purchase intentions, contributing to a deeper understanding of psychological distance and performance expectancy in human–chatbot interaction and digital services. Compared to reactive response strategies, proactive chatbot responses reduce psychological distance, thereby fostering enhanced purchase intentions. As proactive service chatbots exhibit heightened anthropomorphism and human-like representations (Hao et al., 2017; Keidar et al., 2024; Pizzi et al., 2021), they further shorten users’ perceived psychological distance (Park et al., 2024), fostering more favorable evaluations of chatbots (X. Li & Sung, 2021). Likewise, proactive chatbots effectively convey performance expectancy information, elevating users’ anticipations for reliable and consistent service (Lv et al., 2021), which in turn bolsters their purchase intentions. Perceived performance constitutes a crucial factor for customers when assessing the costs and benefits of utilizing chatbots (Gursoy et al., 2019). The proactive model, in comparison to reactive interaction styles, accentuates users’ perceived performance (Keidar et al., 2024). Users’ perception of performance expectancy activates trust mechanisms, ultimately fortifying their behavioral intentions (Mostafa & Kasamani, 2022; Terblanche & Kidd, 2022). The findings resonate with supportive evidence from prior research. Psychological distance and performance expectancy are intertwined mechanisms that significantly impact users’ purchase intentions. The concept of psychological distance is supported by cognitive consistency theory, which posits that individuals strive for harmony between their beliefs and behaviors. Studies have found that when customers perceive chatbots as familiar and trustworthy, the psychological distance diminishes, leading to increased purchase intentions (Zhu et al., 2023b). This is further reinforced by additional findings, which indicate that users’ trust in chatbots significantly influences their perception of the chatbot’s performance, thereby reducing psychological distance and enhancing engagement (A. J. Kim et al., 2021). Some research has asserted that performance expectancy directly influences users’ intentions to utilize chatbots, emphasizing its role in shaping consumer behavior (Romero-Rodríguez et al., 2023). The Unified Theory of Acceptance and Use of Technology (UTAUT) provides a theoretical framework that integrates performance expectancy as a core construct influencing technology adoption. This model has been validated across various studies (Choudhury & Shamszare, 2024; Mostafa & Kasamani, 2022), which confirm the positive correlation between performance expectancy and users’ intentions. The findings suggest that when users believe chatbots will enhance their shopping experience, their purchase intention increases correspondingly.

Thirdly, the exploration of emojis’ moderating role in chatbot service interactions constitutes an emerging research area. This study delves into how emojis moderate the influence of chatbot response strategies on psychological distance, thus contributing preliminary empirical evidence to this nascent field. Notably, the existing literature demonstrates that only a few studies have shown the direct effect of chatbots’ emoji usage in reducing psychological distance (Yu & Zhao, 2024). Other investigations have examined the direct influence of emojis on user engagement, satisfaction, and intention to use in human–chatbot interactions, as well as their indirect effects mediated by perceived factors (D. Liu et al., 2023; Nguyen et al., 2023). Furthermore, this study reinforces the applicability of media richness theory and computer-mediated communication theory in chatbot service interactions, offering new insights into the pivotal role of digital communication tools, particularly emojis, in optimizing user experience. Media richness theory posits that richer media facilitate better communication by providing more cues, which is essential in environments lacking the non-verbal signals that are typical in face-to-face interactions. Emojis serve as visual cues that convey emotions, thereby enriching the textual communication of chatbots and fostering a sense of social presence (Beattie et al., 2020; Y. Li & Shin, 2023; Maar et al., 2023). Computer-mediated communication theory emphasizes the challenges of conveying emotional nuances in text-based communication, where non-verbal cues are absent. Emojis serve as visual representations of emotions, and help bridge the emotional gap that often exists in digital communication, allowing users to interpret the intended emotional tone of messages more accurately, thereby facilitating better interpersonal relationships in digital contexts (Cavalheiro et al., 2022; Daniel & Camp, 2020; Yang & Qian, 2024). Research indicates that the use of emojis can enhance interpersonal impressions and create a more engaging interaction, making users feel more connected to the chatbot (Huda et al., 2024; Jasin et al., 2023). This emotional engagement is crucial for improving user satisfaction and fostering a positive relationship between users and chatbots, ultimately leading to better communication outcomes (K. Jiang et al., 2022; Y. Xu et al., 2022a). Consequently, emojis not only enrich the communicative experience but also align with the principles of computer-mediated communication theory by addressing the inherent limitations of text-based communication.

In addition, this study also provides clues to the negative impacts of the excessive anthropomorphism of chatbots. This study demonstrates that emojis do not significantly moderate the effect of chatbot response strategies on performance expectancy. The use of emojis enhances the perceived cuteness of chatbots. There is a “cuteness effect” across different service types, including emotional and knowledge-based ones (Lv et al., 2022). In knowledge-based tasks, customers tend to have lower performance expectancy for high-cuteness chatbots compared to low-cuteness chatbots (Lv et al., 2021). Additionally, research has highlighted that the use of emojis intensifies the anthropomorphism of chatbots, which can yield a negative impact when employed by brands with low credibility (Nguyen et al., 2023). Moreover, as the level of anthropomorphism in chatbots increases, so does the perceived risk associated with purchasing (Roy & Naidoo, 2021), potentially leading to a reduction in performance expectancy among users. There is evidence suggesting that while anthropomorphism can facilitate transactional outcomes in retail environments, it also leads to a significant increase in offer sensitivity, as consumers shift towards a fair evaluation or negotiation mindset when chatbots become more human-like (Schanke et al., 2021). The anthropomorphization of chatbots, including the inappropriate use of emojis, can result in excessive human-like attributes that may trigger the uncanny valley effect. This phenomenon arises when a robot or chatbot appears almost human but not quite, eliciting feelings of eeriness or discomfort among users (Shin et al., 2023). Research suggests that, while anthropomorphic features can enhance user engagement and satisfaction, excessive use of these traits can provoke negative emotional responses, such as fear or disgust (Blut et al., 2021; Yanxia et al., 2023). For example, studies have emphasized that the degree of anthropomorphism in chatbots significantly influences user intentions, with excessive human-like characteristics potentially eliciting adverse reactions (M. Liu et al., 2024). Furthermore, It has been cautioned that brands should exercise caution in their use of emojis, particularly in contexts where credibility is already low, as this could exacerbate feelings of discomfort (Nguyen et al., 2023). Thus, while emojis can enhance communication, their overuse may inadvertently steer users towards simpler, less anthropomorphic chatbots (Huțul et al., 2024).

### 5.3. Managerial Implications

The managerial implications derived from this study are threefold: firstly, optimizing chatbot response strategies is crucial. The study reveals that chatbot response strategies exert a significant impact on user purchase intentions. Hence, companies should contemplate selecting response strategies tailored to their target user groups during the design and deployment of chatbots. For instance, implementing proactive chatbots in the retail sector (Chien et al., 2024) can enhance user experience and facilitate purchase decisions. These scenarios primarily include pre-sales consultation, after-sales service, intelligent shopping assistance, product recommendations, and order management. However, in scenarios requiring high levels of professional knowledge or significant emotional interaction, the proactive response strategies of chatbots should be used cautiously or avoided, such as in psychological counseling, medical consultation, legal advice, and premium customer service. These scenarios demand high levels of personalization, and proactive service may detract from service quality and consumer satisfaction.

Secondly, strengthening psychological distance management and augmenting performance expectancy experiences are essential. Psychological distance and performance expectancy have been established as pivotal mediators in user decision-making processes. Companies can refine response strategies and interface designs, effectively communicate performance information about products or services, thereby improving psychological distance and performance expectancy, and fostering stronger user purchase intentions and satisfaction. For example, through core functions such as emotion recognition, emotion analysis, and emotion presentation, based on diverse information such as users’ words, tone, and facial expressions, chatbots can intelligently identify users’ emotional states and provide emotional support, engaging in more natural and friendly interactions with users, thereby closing the psychological distance. Simultaneously, chatbots enhance users’ performance expectancy by automatically handling issues such as order inquiries, logistics tracking, and after-sales service, thereby improving service efficiency and quality.

Thirdly, leveraging the effective use of emojis is important. This study underscores that emojis, as a digital communication tool, can not only convey emotions but also significantly enhance user experience. Companies may consider integrating emojis into chatbots or other digital communication avenues to enrich interactions and enhance user engagement. For instance, incorporating suitable emojis into hedonic-oriented chatbots (Yu & Zhao, 2024) or recovery strategies following service failures (D. Liu et al., 2023) could prove beneficial. Specifically, chatbots in service scenarios such as fashion and beauty, food and beverage, as well as leisure and entertainment should appropriately enhance the use of emojis. Emojis can increase interactivity and fun, aiding consumers in better understanding and experiencing product features, thereby enhancing their participation and purchase intention. Conversely, in professional consulting services (such as legal advice, financial consulting, medical services), technical support and maintenance, as well as formal business communications, the use of emojis should be avoided. Communications in these fields require professionalism and solemnity. The use of emojis may undermine this professionalism and even cause consumer misunderstanding or dissatisfaction.

### 5.4. Limitations and Future Research

Firstly, there is a lack of real human–chatbot interaction experiments. This study simulated user interactions with chatbots using image stimuli instead of actual human–chatbot interaction experiments. This method may not fully reflect real user experiences and behavioral responses. Future research could validate the external validity of the results through actual user interactions with chatbots or interactive experiments conducted in a laboratory setting. Secondly, the enhancement of proactive response effects. Proactive chatbots can provide more feedback, potentially increasing the amount of information acquired by users and thereby enhancing the user experience. At the same time, proactive responses may also result in longer interaction times between chatbots and users, thus increasing psychological distance. Future research could delve deeper into exploring the combined effects of different response strategies on interaction time, information volume, and psychological distance, aiming to find a more optimized balance. Thirdly, the moderating effect of emojis on the relationship between chatbot response strategies and performance expectancy is not significant (i.e., H5 is not supported). The reasons for this are unclear, and future research with scientific designs is needed to uncover the underlying mechanisms involved. Emojis carry rich cultural attributes and meanings. For instance, in some cultural contexts, giving a thumbs-up can be a provocative act, which stands in stark contrast to its universally recognized meanings of “approval” or “good”. Furthermore, in certain cultures, overusing the “smiley face” may be perceived as dishonest or used to conceal genuine emotions. Therefore, future research should focus on the cultural dependency of emojis and compare the impact of emojis across different cultures on the results of this study. Furthermore, this study did not consider the different consumption motivations for various products, such as utilitarian and hedonic motivations (Hu et al., 2023), and thus failed to explore whether there are differences in research results under different consumption motivations. Future studies can further segment product types and consumption motivations to gain a more comprehensive understanding of the impact mechanism of chatbot response strategies on users’ purchase intention. 

## Figures and Tables

**Table 1:** Representative literature on interaction strategies of chatbots in customer service.

Perspectives	Sources	Methods/Experimental Design	Findings
Interaction modes	(Keidar et al., 2024)	Two (interaction modes: proactive vs. reactive) × two (types of user populations: with vs. without technological backgrounds)	The proactive mode can increase the user’s situation awareness, improve the user’s performance, and reduce the users’ perceived workload in the reactive mode.
Social manner and expressive behavior	(Chien et al., 2024)	Two (social manner: proactive vs. reactive) × two (expressive behavior: intimate vs. impassive)	When a social humanoid robot behaves in a more proactive manner and is perceived as more intimate, users are more willing to trust the robot, thereby increasing their intention to use.
Politeness strategy	(Song et al., 2023)	Two (politeness strategies: appreciation vs. apology) × two (time pressure: high vs. low)	Following a service failure, the appreciation politeness strategy enhances consumers’ post-recovery satisfaction more effectively than the apology strategy. This effect is mediated by face concern and moderated by time pressure.
Interaction style	(De Cicco et al., 2020)	Two (visual cues: avatar presence vs. avatar absence) × two (interaction styles: social-oriented vs. task-oriented)	Applying a social-oriented interaction style increases users’ perception of social presence, while an insignificant effect was found for avatar presence.
Conversation initiation	(Pizzi et al., 2021)	Two (assistant type: anthropomorphic vs. non-anthropomorphic) × two (assistant initiation: user- vs. system-initiated)	An automatically activated non-anthropomorphic digital assistant yields higher satisfaction and user experience compared to a human-like, consumer-activated one.
Conversational style	(Roy & Naidoo, 2021)	Two (time orientation: present vs. future) × two (anthropomorphic conversation style: warm vs. competent)	For a present orientation, a warm chatbot conversation boosts attitude and purchase intention through brand warmth. For a future orientation, a competent chatbot conversation enhances these outcomes via brand competence.
Communication strategy	(Tsai et al., 2021)	Two (social presence communication: high vs. low) × two (anthropomorphic vs. non-anthropomorphic bot profile)	The impact of chatbots’ high social presence communication on consumer engagement is mediated by perceived parasocial interaction and dialog. Moreover, anthropomorphic profile design enhances these effects through psychological mediators.
Communication style	(Shin et al., 2023)	(i) Two (humor: no humor vs. humor) × two (agent type: chatbot vs. human) (ii) humor style (affiliative vs. aggressive)	Humor used by chatbots boosts service satisfaction, with this effect mediated by increased anthropomorphism and interestingness of the interactions. Socially appropriate (i.e., affiliative) humor, compared to inappropriate (i.e., aggressive) humor, leads to greater service satisfaction.
Communication style	(Maar et al., 2023)	Two (communication style: high vs. low social orientation) × two (customer generation: generation X [GenX] vs. generation Z [GenZ]) × two (service context: restaurant vs. medical)	GenZ views chatbots more positively than GenX, due to greater perceptions of warmth and competence. While GenZ’s attitudes toward chatbots are similar regardless of social orientation, GenX prefers chatbots with high social orientation, seeing them as warmer and more favorable.
Language style	(Y. Huang & Gursoy, 2024)	Two (language style: abstract language vs. concrete language) × two (decision-making journey stage: informational stage vs. transactional stage)	During the informational stage, an abstract language style in chatbots strongly influences service satisfaction through emotional support. In the transactional stage, a concrete language style in chatbots strongly impacts service satisfaction through informational support.
Language style	(M. Li & Wang, 2023)	Two (chatbot language style: formal vs. informal) × two (brand affiliation: customer vs. non-customer)	Informal chatbot language increases customers’ continued usage intention and brand attitude through parasocial interaction. Brand affiliation moderates this effect, weakening it for those with no prior brand relationship.
Language concreteness	(Jiménez-Barreto et al., 2023).	Two (language concreteness: high vs. low) × three (conversation stage: opening vs. query/response vs. closing)	High chatbot language concreteness enhances perceived chatbot and consumer competence, satisfaction, and perceived shopping efficiency.
Service scripts	(Sands et al., 2021)	Two (service interaction: human vs. chatbot) × two (service script: education vs. entertainment)	When using an education script, human service agents significantly outperform chatbots in satisfaction and purchase intention, with emotion and rapport fully mediating these effects.

**Table 2:** ANOVA results of Study 2.

Variables	Experimental Groups		F	*p*
	Proactive	Reactive		
Psychological distance	4.853	4.054	94.730	0.000
Performance expectancy	4.787	3.986	78.902	0.000
Purchase intention	5.186	4.495	83.471	0.000

**Table 3:** Bootstrap test for mediating effects (Study 2).

Effects	Mediating Variables	Effects	se	t	*p*	95% CI
Direct effect	—	0.237	0.082	2.900	0.004	[0.075, 0.398]
Mediating effects	Psychological distance	0.221	0.086	—	—	[0.048, 0.388]
	Performance expectancy	0.234	0.078	—	—	[0.086, 0.398]

**Table 4:** ANOVA results of Study 3.

Variables	Experimental Groups with Emojis				Experimental Groups without Emojis			
	Proactive	Reactive	F	*p*	Proactive	Reactive	F	*p*
Psychological distance	5.639	3.961	440.068	0.000	4.930	3.667	184.091	0.000
Performance expectancy	4.877	3.481	272.934	0.000	4.829	3.661	143.639	0.000
Purchase intention	5.881	4.251	429.216	0.000	4.952	3.811	161.106	0.000

**Table 5:** Moderating role of emojis in the relationship between RS and PD.

	Type III Sums of Squares	df	Mean Square	F	Sig.
Corrected Model	183.534	3	61.178	214.74	0.000
Response Strategies (RS)	163.236	1	163.236	572.972	0.000
Emojis	19.012	1	19.012	66.733	0.000
RS × Emojis	3.251	1	3.251	11.410	0.001
Error	84.898	298	0.285		

**Table 6:** Moderating role of emojis in the relationship between RS and PE.

	Type III Sums of Squares	df	Mean Square	F	Sig.
Corrected Model	125.415	3	41.805	132.918	0.000
Response Strategies (RS)	124.032	1	124.032	394.359	0.000
Emojis	0.333	1	0.333	1.060	0.304
RS × Emojis	0.985	1	0.985	3.130	0.078
Error	93.725	298	0.315		

## Data Availability

The data are available upon request from the corresponding author.

## References

[B1-behavsci-15-00117] Abdallah W., Harraf A., Mosusa O., Sartawi A. (2023). Investigating factors impacting customer acceptance of artificial intelligence chatbot: Banking sector of Kuwait. International Journal of Applied Research in Management and Economics.

[B2-behavsci-15-00117] Adam M., Wessel M., Benlian A. (2021). AI-based chatbots in customer service and their effects on user compliance. Electronic Markets.

[B3-behavsci-15-00117] Aslam F. (2023). The Impact of artificial intelligence on chatbot technology: A study on the current advancements and leading innovations. European Journal of Technology.

[B4-behavsci-15-00117] Azalan N. S., Mokhtar M. M., Abdul Karim A. H. (2022). Modelling e-Zakat acceptance among Malaysian: An application of UTAUT model during Covid19 pandemic. International Journal of Academic Research in Business and Social Sciences.

[B5-behavsci-15-00117] Bai Q., Dan Q., Mu Z., Yang M. (2019). A systematic review of emoji: Current research and future perspectives. Frontiers in Psychology.

[B6-behavsci-15-00117] Beattie A., Edwards A. P., Edwards C. (2020). A bot and a smile: Interpersonal impressions of chatbots and humans using emoji in computer-mediated communication. Communication Studies.

[B7-behavsci-15-00117] Blazevic V., Sidaoui K. (2022). The TRISEC framework for optimizing conversational agent design across search, experience and credence service contexts. Journal of Service Management.

[B8-behavsci-15-00117] Blut M., Wang C., Wünderlich N. V., Brock C. (2021). Understanding anthropomorphism in service provision: A meta-analysis of physical robots, chatbots, and other AI. Journal of the Academy of Marketing Science.

[B9-behavsci-15-00117] Buyukgoz S., Grosinger J., Chetouani M., Saffiotti A. (2022). Two ways to make your robot proactive: Reasoning about human intentions or reasoning about possible futures. Frontiers in Robotics and AI.

[B10-behavsci-15-00117] Cai D., Li H., Law R. (2022). Anthropomorphism and OTA chatbot adoption: A mixed methods study. Journal of Travel & Tourism Marketing.

[B11-behavsci-15-00117] Cavalheiro B. P., Prada M., Rodrigues D. L., Lopes D., Garrido M. V. (2022). Evaluating the adequacy of emoji use in positive and negative messages from close and distant senders. Cyberpsychology, Behavior, and Social Networking.

[B12-behavsci-15-00117] Chen Q., Lu Y., Gong Y., Xiong J. (2023). Can AI chatbots help retain customers? Impact of AI service quality on customer loyalty. Internet Research.

[B13-behavsci-15-00117] Chen S., Li X., Liu K., Wang X. (2023). Chatbot or human? The impact of online customer service on consumers’ purchase intentions. Psychology & Marketing.

[B14-behavsci-15-00117] Chien S.-Y., Lin Y.-L., Chang B.-F. (2024). The effects of intimacy and proactivity on trust in human-humanoid robot interaction. Information Systems Frontiers.

[B15-behavsci-15-00117] Choudhury A., Shamszare H. (2024). The impact of performance expectancy, workload, risk, and satisfaction on trust in ChatGPT: Cross-sectional survey analysis. JMIR Human Factors.

[B16-behavsci-15-00117] Chung S. I., Han K.-H. (2022). Consumer perception of chatbots and purchase intentions: Anthropomorphism and conversational relevance. International Journal of Advanced Culture Technology.

[B17-behavsci-15-00117] Crolic C., Thomaz F., Hadi R., Stephen A. T. (2021). Blame the bot: Anthropomorphism and anger in customer–chatbot interactions. Journal of Marketing.

[B18-behavsci-15-00117] Daniel T. A., Camp A. L. (2020). Emojis affect processing fluency on social media. Psychology of Popular Media.

[B19-behavsci-15-00117] De Cicco R., Silva S. C., Alparone F. R. (2020). Millennials’ attitude toward chatbots: An experimental study in a social relationship perspective. International Journal of Retail & Distribution Management.

[B20-behavsci-15-00117] Eiskjær S., Pedersen C. F., Skov S. T., Andersen M. Ø (2023). Usability and performance expectancy govern spine surgeons’ use of a clinical decision support system for shared decision-making on the choice of treatment of common lumbar degenerative disorders. Frontiers in Digital Health.

[B21-behavsci-15-00117] El-Shihy D., Abdelraouf M., Hegazy M., Hassan N. (2024). The influence of AI chatbots in fintech services on customer loyalty within the banking industry. Future of Business Administration.

[B22-behavsci-15-00117] Erle T. M., Schmid K., Goslar S. H., Martin J. D. (2022). Emojis as social information in digital communication. Emotion.

[B23-behavsci-15-00117] Fournier-Tombs E., McHardy J. (2023). A medical ethics framework for conversational artificial intelligence. Journal of Medical Internet Research.

[B24-behavsci-15-00117] Ghosh S., Ness S., Salunkhe S. (2024). The role of AI enabled chatbots in omnichannel customer service. Journal of Engineering Research and Reports.

[B25-behavsci-15-00117] Gnewuch U., Morana S., Hinz O., Kellner R., Maedche A. (2023). More than a bot? The impact of disclosing human involvement on customer interactions with hybrid service agents. Information Systems Research.

[B26-behavsci-15-00117] Grosinger J., Pecora F., Saffiotti A. (2019). Robots that maintain equilibrium: Proactivity by reasoning about user intentions and preferences. Pattern Recognition Letters.

[B27-behavsci-15-00117] Gursoy D., Chi O. H., Lu L., Nunkoo R. (2019). Consumers acceptance of artificially intelligent (AI) device use in service delivery. International Journal of Information Management.

[B28-behavsci-15-00117] Han E., Yin D., Zhang H. (2023). Bots with feelings: Should AI agents express positive emotion in customer service?. Information Systems Research.

[B29-behavsci-15-00117] Hao M., Cao W., Wu M., Liu Z., She J., Chen L., Zhang R. (2017). Proposal of initiative service model for service robot. CAAI Transactions on Intelligence Technology.

[B30-behavsci-15-00117] Haupt M., Rozumowski A., Freidank J., Haas A. (2023). Seeking empathy or suggesting a solution? Effects of chatbot messages on service failure recovery. Electronic Markets.

[B31-behavsci-15-00117] Hu L., Filieri R., Acikgoz F., Zollo L., Rialti R. (2023). The effect of utilitarian and hedonic motivations on mobile shopping outcomes. A cross-cultural analysis. International Journal of Consumer Studies.

[B32-behavsci-15-00117] Huang M.-H., Rust R. T. (2021). Engaged to a robot? The role of AI in service. Journal of Service Research.

[B33-behavsci-15-00117] Huang Y., Gursoy D. (2024). Customers’ online service encounter satisfaction with chatbots: Interaction effects of language style and decision-making journey stage. International Journal of Contemporary Hospitality Management.

[B34-behavsci-15-00117] Huda N. U., Sahito S. F., Gilal A. R., Abro A., Alshanqiti A., Alsughayyir A., Palli A. S. (2024). Impact of contradicting subtle emotion cues on large language models with various prompting techniques. International Journal of Advanced Computer Science and Applications (IJACSA).

[B35-behavsci-15-00117] Huțul T.-D., Popescu A., Karner-Huțuleac A., Holman A. C., Huțul A. (2024). Who’s willing to lay on the virtual couch? Attitudes, anthropomorphism and need for human interaction as factors of intentions to use chatbots for psychotherapy. Counselling and Psychotherapy Research.

[B36-behavsci-15-00117] Ischen C., Araujo T., Van Noort G., Voorveld H., Smit E. (2020). “I am here to assist you today”: The role of entity, interactivity and experiential perceptions in chatbot persuasion. Journal of Broadcasting & Electronic Media.

[B37-behavsci-15-00117] Jasin J., Ng H. T., Atmosukarto I., Iyer P., Osman F., Wong P. Y. K., Pua C. Y., Cheow W. S. (2023). The implementation of chatbot-mediated immediacy for synchronous communication in an online chemistry course. Education and Information Technologies.

[B38-behavsci-15-00117] Jiang H., Cheng Y., Yang J., Gao S. (2022). AI-powered chatbot communication with customers: Dialogic interactions, satisfaction, engagement, and customer behavior. Computers in Human Behavior.

[B39-behavsci-15-00117] Jiang K., Qin M., Li S. (2022). Chatbots in retail: How do they affect the continued use and purchase intentions of Chinese consumers?. Journal of Consumer Behaviour.

[B40-behavsci-15-00117] Jiménez-Barreto J., Rubio N., Molinillo S. (2023). How chatbot language shapes consumer perceptions: The role of concreteness and shared competence. Journal of Interactive Marketing.

[B41-behavsci-15-00117] Kang J.-W., Namkung Y. (2019). The information quality and source credibility matter in customers’ evaluation toward food O2O commerce. International Journal of Hospitality Management.

[B42-behavsci-15-00117] Keidar O., Parmet Y., Olatunji S. A., Edan Y. (2024). Comparison of proactive and reactive interaction modes in a mobile robotic telecare study. Applied Ergonomics.

[B43-behavsci-15-00117] Kim A. J., Yang J., Jang Y., Baek J. S. (2021). Acceptance of an informational antituberculosis chatbot among korean adults: Mixed methods research. JMIR mHealth and uHealth.

[B44-behavsci-15-00117] Kim J., Lucas A. F. (2024). Perceptions and acceptance of cashless gambling technology: An empirical study of U.S. and Australian consumers. Cornell Hospitality Quarterly.

[B45-behavsci-15-00117] Klein K., Martinez L. F. (2023). The impact of anthropomorphism on customer satisfaction in chatbot commerce: An experimental study in the food sector. Electronic Commerce Research.

[B46-behavsci-15-00117] Li B., Yao R., Nan Y. (2023). How do friendship artificial intelligence chatbots (FAIC) benefit the continuance using intention and customer engagement?. Journal of Consumer Behaviour.

[B47-behavsci-15-00117] Li D., Liu C., Xie L. (2022). How do consumers engage with proactive service robots? The roles of interaction orientation and corporate reputation. International Journal of Contemporary Hospitality Management.

[B48-behavsci-15-00117] Li M., Wang R. (2023). Chatbots in e-commerce: The effect of chatbot language style on customers’ continuance usage intention and attitude toward brand. Journal of Retailing and Consumer Services.

[B49-behavsci-15-00117] Li X., Sung Y. (2021). Anthropomorphism brings us closer: The mediating role of psychological distance in User–AI assistant interactions. Computers in Human Behavior.

[B50-behavsci-15-00117] Li Y., Shin H. (2023). Should a luxury brand’s chatbot use emoticons? Impact on brand status. Journal of Consumer Behaviour.

[B51-behavsci-15-00117] Liang H.-Y., Chu C.-Y., Lin J.-S.C. (2020). Engaging customers with employees in service encounters: Linking employee and customer service engagement behaviors through relational energy and interaction cohesion. Journal of Service Management.

[B52-behavsci-15-00117] Liu D., Lv Y., Huang W. (2023). How do consumers react to chatbots’ humorous emojis in service failures. Technology in Society.

[B53-behavsci-15-00117] Liu M., Yang Y., Ren Y., Jia Y., Ma H., Luo J., Fang S., Qi M., Zhang L. (2024). What influences consumer AI chatbot use intention? An application of the extended technology acceptance model. Journal of Hospitality and Tourism Technology.

[B54-behavsci-15-00117] Liu S. Q., Vakeel K. A., Smith N. A., Alavipour R. S., Wei C., Wirtz J. (2024). AI concierge in the customer journey: What is it and how can it add value to the customer?. Journal of Service Management.

[B55-behavsci-15-00117] Lv X., Liu Y., Luo J., Liu Y., Li C. (2021). Does a cute artificial intelligence assistant soften the blow? The impact of cuteness on customer tolerance of assistant service failure. Annals of Tourism Research.

[B56-behavsci-15-00117] Lv X., Luo J., Liang Y., Liu Y., Li C. (2022). Is cuteness irresistible? The impact of cuteness on customers’ intentions to use AI applications. Tourism Management.

[B57-behavsci-15-00117] Maar D., Besson E., Kefi H. (2023). Fostering positive customer attitudes and usage intentions for scheduling services via chatbots. Journal of Service Management.

[B58-behavsci-15-00117] Majeed S., Kim W. G. (2024). Antecedents and consequences of conceptualizing online hyperconnected brand selection. Journal of Consumer Marketing.

[B59-behavsci-15-00117] Matini A., Lekata S., Kabaso B. (2024). The effects of stress and chatbot services usage on customer intention for purchase on E-commerce sites. International Journal on Data Science and Technology.

[B60-behavsci-15-00117] Meyer N., Weiger W., Hammerschmidt M. (2022). Trust me, I’m a bot—Repercussions of chatbot disclosure in different service frontline settings. Journal of Service Management.

[B61-behavsci-15-00117] Milenia Ayukharisma M. A., Budi Santoso D. (2024). Examining healthcare profesional’s acceptance of electronic medical records system using extended UTAUT2. Buana Information Technology and Computer Sciences (BIT and CS).

[B62-behavsci-15-00117] Mostafa R. B., Kasamani T. (2022). Antecedents and consequences of chatbot initial trust. European Journal of Marketing.

[B63-behavsci-15-00117] Myin M. T., Watchravesringkan K. (2024). Investigating consumers’ adoption of AI chatbots for apparel shopping. Journal of Consumer Marketing.

[B64-behavsci-15-00117] Nguyen M., Casper Ferm L.-E., Quach S., Pontes N., Thaichon P. (2023). Chatbots in frontline services and customer experience: An anthropomorphism perspective. Psychology & Marketing.

[B65-behavsci-15-00117] Oh Y. J., Zhang J., Fang M.-L., Fukuoka Y. (2021). A systematic review of artificial intelligence chatbots for promoting physical activity, healthy diet, and weight loss. International Journal of Behavioral Nutrition and Physical Activity.

[B66-behavsci-15-00117] Olk S., Tscheulin D. K., Lindenmeier J. (2021). Does it pay off to smile even it is not authentic? Customers’ involvement and the effectiveness of authentic emotional displays. Marketing Letters.

[B67-behavsci-15-00117] Otterbring T., Arsenovic J., Samuelsson P., Malodia S., Dhir A. (2024). Going the extra mile, now or after a while: The impact of employee proactivity in retail service encounters on customers’ shopping responses. British Journal of Management.

[B68-behavsci-15-00117] Paraskevi G., Saprikis V., Avlogiaris G. (2023). Modeling nonusers’ behavioral intention towards mobile chatbot adoption: An extension of the UTAUT2 model with mobile service quality determinants. Human Behavior and Emerging Technologies.

[B69-behavsci-15-00117] Park G., Chung J., Lee S. (2024). Human vs. machine-like representation in chatbot mental health counseling: The serial mediation of psychological distance and trust on compliance intention. Current Psychology.

[B70-behavsci-15-00117] Pillai R., Ghanghorkar Y., Sivathanu B., Algharabat R., Rana N. P. (2024). Adoption of artificial intelligence (AI) based employee experience (EEX) chatbots. Information Technology & People.

[B71-behavsci-15-00117] Pizzi G., Scarpi D., Pantano E. (2021). Artificial intelligence and the new forms of interaction: Who has the control when interacting with a chatbot?. Journal of Business Research.

[B72-behavsci-15-00117] Ramesh A., Chawla V. (2022). Chatbots in marketing: A literature review using morphological and co-occurrence analyses. Journal of Interactive Marketing.

[B73-behavsci-15-00117] Riordan M. A. (2017). Emojis as tools for emotion work: Communicating affect in text messages. Journal of Language and Social Psychology.

[B74-behavsci-15-00117] Romero-Rodríguez J.-M., Ramírez-Montoya M.-S., Buenestado-Fernández M., Lara-Lara F. (2023). Use of ChatGPT at university as a tool for complex thinking: Students’ perceived usefulness. Journal of New Approaches in Educational Research.

[B75-behavsci-15-00117] Roy R., Naidoo V. (2021). Enhancing chatbot effectiveness: The role of anthropomorphic conversational styles and time orientation. Journal of Business Research.

[B76-behavsci-15-00117] Sands S., Ferraro C., Campbell C., Tsao H.-Y. (2021). Managing the human–chatbot divide: How service scripts influence service experience. Journal of Service Management.

[B77-behavsci-15-00117] Schanke S., Burtch G., Ray G. (2021). Estimating the impact of “humanizing” customer service chatbots. Information Systems Research.

[B78-behavsci-15-00117] Schelble B. G., Flathmann C., McNeese N. J., O’Neill T., Pak R., Namara M. (2023). Investigating the effects of perceived teammate artificiality on human performance and cognition. International Journal of Human–Computer Interaction.

[B79-behavsci-15-00117] Shin H., Bunosso I., Levine L. R. (2023). The influence of chatbot humour on consumer evaluations of services. International Journal of Consumer Studies.

[B80-behavsci-15-00117] Song M., Zhang H., Xing X., Duan Y. (2023). Appreciation vs. apology: Research on the influence mechanism of chatbot service recovery based on politeness theory. Journal of Retailing and Consumer Services.

[B81-behavsci-15-00117] Terblanche N., Kidd M. (2022). Adoption factors and moderating effects of age and gender that influence the intention to use a non-directive reflective coaching chatbot. SAGE Open.

[B82-behavsci-15-00117] Tian W., Ge J., Zhao Y., Zheng X. (2024). AI Chatbots in Chinese higher education: Adoption, perception, and influence among graduate students—An integrated analysis utilizing UTAUT and ECM models. Frontiers in Psychology.

[B83-behavsci-15-00117] Trautmann S. T. (2019). Distance from a distance: The robustness of psychological distance effects. Theory and Decision.

[B84-behavsci-15-00117] Trope Y., Liberman N. (2000). Temporal construal and time-dependent changes in preference. Journal of Personality and Social Psychology.

[B85-behavsci-15-00117] Trzebiński W., Claessens T., Buhmann J., De Waele A., Hendrickx G., Van Damme P., Daelemans W., Poels K. (2023). The effects of expressing empathy/autonomy support using a COVID-19 vaccination chatbot: Experimental study in a sample of Belgian adults. JMIR Formative Research.

[B86-behavsci-15-00117] Tsai W.-H. S., Liu Y., Chuan C.-H. (2021). How chatbots’ social presence communication enhances consumer engagement: The mediating role of parasocial interaction and dialogue. Journal of Research in Interactive Marketing.

[B87-behavsci-15-00117] Tsaur S.-H., Yen C.-H. (2019). Service redundancy in fine dining: Evidence from Taiwan. International Journal of Contemporary Hospitality Management.

[B88-behavsci-15-00117] Vu H. N., Nguyen T. K. C. (2022). The impact of AI chatbot on customer willingness to pay: An empirical investigation in the hospitality industry. Journal of Trade Science.

[B89-behavsci-15-00117] Wang H., Gupta S., Singhal A., Muttreja P., Singh S., Sharma P., Piterova A. (2022). An artificial intelligence chatbot for young people’s sexual and reproductive health in India (SnehAI): Instrumental case study. Journal of Medical Internet Research.

[B90-behavsci-15-00117] Wu R., Chen J., Lu Wang C., Zhou L. (2022). The influence of emoji meaning multipleness on perceived online review helpfulness: The mediating role of processing fluency. Journal of Business Research.

[B91-behavsci-15-00117] Xiao Z., Zhou M. X., Liao Q. V., Mark G., Chi C., Chen W., Yang H. (2020). Tell me about yourself: Using an AI-powered chatbot to conduct conversational surveys with open-ended questions. ACM Transactions on Computer-Human Interaction.

[B92-behavsci-15-00117] Xie T., Pentina I., Hancock T. (2023). Friend, mentor, lover: Does chatbot engagement lead to psychological dependence?. Journal of Service Management.

[B93-behavsci-15-00117] Xu F., Niu W., Li S., Bai Y. (2020). The mechanism of word-of-mouth for tourist destinations in crisis. SAGE Open.

[B94-behavsci-15-00117] Xu Y., Zhang J., Chi R., Deng G. (2022a). Enhancing customer satisfaction with chatbots: The influence of anthropomorphic communication styles and anthropomorphised roles. Nankai Business Review International.

[B95-behavsci-15-00117] Xu Y., Zhang J., Deng G. (2022b). Enhancing customer satisfaction with chatbots: The influence of communication styles and consumer attachment anxiety. Frontiers in Psychology.

[B96-behavsci-15-00117] Yang K., Qian S. (2024). Your smiling face is impolite to me: A study of the smiling face emoji in Chinese computer-mediated communication. Social Science Computer Review.

[B97-behavsci-15-00117] Yanxia C., Shijia Z., Yuyang X. (2023). A meta-analysis of the effect of chatbot anthropomorphism on the customer journey. Marketing Intelligence & Planning.

[B98-behavsci-15-00117] Yoon J., Yu H. (2022). Impact of customer experience on attitude and utilization intention of a restaurant-menu curation chatbot service. Journal of Hospitality and Tourism Technology.

[B99-behavsci-15-00117] Youn K., Cho M. (2023). Business types matter: New insights into the effects of anthropomorphic cues in AI chatbots. Journal of Services Marketing.

[B100-behavsci-15-00117] Yu S., Zhao L. (2024). Emojifying chatbot interactions: An exploration of emoji utilization in human-chatbot communications. Telematics and Informatics.

[B101-behavsci-15-00117] Zhou A., Tsai W.-H. S., Men L. R. (2024). Optimizing AI social chatbots for relational outcomes: The effects of profile design, communication strategies, and message framing. International Journal of Business Communication.

[B102-behavsci-15-00117] Zhu Y., Zhang J., Wu J. (2023a). Who did what and when? The effect of chatbots’ service recovery on customer satisfaction and revisit intention. Journal of Hospitality and Tourism Technology.

[B103-behavsci-15-00117] Zhu Y., Zhang R., Zou Y., Jin D. (2023b). Investigating customers’ responses to artificial intelligence chatbots in online travel agencies: The moderating role of product familiarity. Journal of Hospitality and Tourism Technology.

